# Interplay between Discrete Emotions and Preventive Behavior in Health Crises: Big Data Analysis of COVID-19

**DOI:** 10.3390/ijerph192416407

**Published:** 2022-12-07

**Authors:** Huiyun Zhu

**Affiliations:** School of Management Science and Engineering, Nanjing University of Information Science & Technology, Nanjing 210044, China; zhy@nuist.edu.cn

**Keywords:** preventive behavior, emotion analysis, Granger causality analysis, impulse response functions, big data analytics, social media

## Abstract

Understanding the interplay between discrete emotions and COVID-19 prevention behaviors will help healthcare professionals and providers to implement effective risk communication and effective risk decision making. This study analyzes data related to COVID-19 posted by the American public on Twitter and identifies three discrete negative emotions (anger, anxiety, and sadness) of the public from massive text data. Next, econometric analyses (i.e., the Granger causality test and impulse response functions) are performed to evaluate the interplay between discrete emotions and preventive behavior based on emotional time series and Google Shopping Trends time series, representing public preventive behavior. Based on the textual analysis of tweets from the United States, the following conclusions are drawn: Anger is a Granger cause of preventive behavior and has a slightly negative effect on the public’s preventive behavior. Anxiety is a Granger cause of preventive behavior and has a positive effect on preventive behavior. Furthermore, preventive behavior is a Granger cause of anxiety and has a negative and lagging effect on anxiety. Exploring how discrete emotions, such as anger and anxiety, affect preventive behaviors will effectively demonstrate how discrete emotions play qualitatively different roles in promoting preventive behaviors. Moreover, understanding the impact of preventive behaviors on discrete emotions is useful for better risk communication.

## 1. Introduction

The COVID-19 pandemic has brought unbearably huge losses to countries around the world. To control the pandemic, experts from the public health department have advised the public to take preventive measures, such as reducing public gatherings, washing their hands frequently, and wearing masks. However, the success in mitigating public health risks depends on the extent of cooperation of the public [1,2,3]. Given the critical importance of preventive health behaviors in mitigating the spread of COVID-19, there is a dire need to understand patterns of behavioral engagement in daily life over time [4].

Global aspects of emotion have been central to psychosocial theories of health and health behavior for several decades [5]. Dominant theories suggest that emotions influence judgments and decision making, which, in turn, could influence behavioral outcomes [6,7,8]. To discuss the specific relationship between emotion and decision making, the valence-based approach divides emotion into positive emotion and negative emotion, arguing that positive-emotion decision makers tend to maintain a current situation and prefer risk avoidance, while negative-emotion decision makers prefer risk-seeking [9,10]. That is to say, emotions with the same valence have the same direction of influence on risk decision making [11]. However, Lerner et al. [12] find that, although the valence of anger and fear are both negative, there are strikingly opposite effects for anger and fear on risk judgments and policy preferences: while fear increases risk estimates and plans for precautionary measures, anger does the opposite. Other studies have found that although anger and happiness have opposite valence, they will promote individuals to report similar judgment [13]. These studies suggest that emotions have properties beyond valence, and valence-based approaches cannot detect the different influences of discrete emotions on risk decision making.

The influences of discrete emotions on risk decision making should be discussed from the perspective of emotion specificity. According to the Appraisal Tendency Framework (ATF), the influence of emotion on decision making is reflected in the specific type of emotion, rather than the valence [6,7,11]. Discrete emotions of the same valence may have distinct, yet predictable, influences on decision processes [14]. The ATF proposes a model of emotion-specific influences on judgment and choice, and provides a useful theoretical perspective for analyzing the impact of discrete emotions [7]. Previous studies have analyzed the different effects of different discrete emotions, such as fear and anger, on risk perception and risk judgment [12,15].

There is no doubt that, during public health crises, emotions affect the preventive behaviors the public adopts, just as preventive behaviors affect the emotions the public experiences. However, previous studies have mostly used cross-sectional survey data to analyze the relationship between emotion and preventive behavior [15,16]. Cross-sectional data can analyze correlations between emotions and preventive behaviors, but these correlations by themselves do not identify any direction of influence. A causal relationship between emotion and preventive behavior is hard to generalize. For example, fear may increase an individual’s risk perception of disease. At the same time, however, it is also plausible that those who perceive greater risk may be highly fearful [15]. Therefore, it is of great interest to design and obtain longitudinal data to analyze the complex interplay of discrete emotions and preventive behavior.

The emotions generated by people after disasters are generally negative emotions. The COVID-19 pandemic has had a major impact on the public’s mental health, creating negative emotions, such as anxiety, sadness, anger, and so on [17,18,19]. Not only can negative emotions influence behavioral outcomes through risk perception, but negative emotions can also directly motivate preventive behaviors during a disaster [15,20]. When a disaster occurs, social media users generate massive amounts of data on social media platforms such as Facebook and Twitter [21]. The information posted by the public reflects their positions and emotions [22]. This paper identifies three of the most common negative emotions (anger, anxiety and sadness) during the COVID-19 pandemic from public tweets.

Public preventive behavior can be measured by the trend of purchases of protective equipment. In today’s internet age, the public tend to shop using shopping sites such as Amazon. Especially during the COVID-19 pandemic, more people chose to shop online due to travel restrictions and fear of indoor gatherings. Therefore, Google Shopping Trends data can be analyzed to identify trends in the public’s purchase of protective items, such as hand sanitizers, and be used as a measure of public preventive behavior.

Thus, this paper identifies the daily emotions of the public from the massive text data related to COVID-19 on social media and forms an emotional time series. At the same time, the time series of public protection behaviors are obtained through Google Trends. Additionally, on this basis, this paper assesses the Granger causal relationships between three discrete negative emotions (anxiety, anger, and sadness) and pandemic-preventive behaviors. The following two research questions are answered through the Granger causality test and impulse response functions (IRFs):

Q1: What are the Granger causal relationships between discrete emotions and protection behavior?

Q2: To what extent do discrete emotions affect protection behavior, and vice versa?

## 2. Relevant Theory and Literature

### 2.1. ATF

Many scholars have explored the relationship between emotions and decision making and proposed various theories, such as the affect-as-information theory, the affect heuristic theory, and the previously mentioned ATF theory. The affect-as-information theory and the affect heuristic theory are valence-based theories [23,24]. The valence-based theories of affect influencing judgment and choice contrast with the effects of positive versus negative feeling states of decision making [10,25]. However, these methods do not clarify whether and when discrete emotions of the same valence influence judgments in different ways [7,12]. The ATF theory is an appraisal-based approach that believes that the influence of emotion on decision making is reflected in the specific type of emotion, rather than its valence [6,7,11].

The ATF theory maintains that each discrete emotion, such as anger and anxiety, can be defined by cognitive appraisal dimensions and core appraisal themes which determine the type of emotion and its influence on decision making [13]. For example, fear (defined by great uncertainty and situational control) predicts pessimistic assessments, and can trigger problem-solving or problem-avoidance behaviors to prevent feared events or situations from occurring [6,7,15]. Anger (defined by certainty and individual control) predicts optimistic assessments and could be negatively associated with risk perception [6,7]. The ATF proposes a theoretical link between the cognitive, affective, and behavioral components of human–stimulus interactions by considering the impact of emotions on these interactions [6,7,26].

The ATF theory is used in many application fields. One stream of ATF research addresses emotional carry-over effects on the assessment of risk [13,15,26,27,28,29,30,31,32]. The ATF is presented as a useful means of integrating cognitive and affective approaches to risk perception. Drawing on the ATF, researchers examine the effects of anger, anxiety, and sadness, among others, on risk perception [13,15,26,27,28,29,30,31,32]. Researchers have also used the ATF to discover the differential effects of discrete emotions on moral judgments [33,34]. The ATF sets the stage for predictions about how distinct emotions prioritize specific sociomoral concerns, and thus promote different moral judgments [33]. Drawing on the ATF, researchers examine the effects of disgust and sadness on routine economic transactions [13]. Disgust carries over to normatively unrelated economic decisions, reducing selling and choice prices; however, sadness reduces selling prices but increases choice prices [35].

### 2.2. The Effect of Discrete Emotions on Decision Making during a Crisis

Disaster events are often sudden, unpredictable and serious hazards, resulting in a kind of pressure that will lead to different degrees of negative emotions. Based on the ATF and other theories, researchers analyze the effects of negative emotions, such as fear, anger, anxiety, disgust, and sadness, on risk perception and preventive actions.

Fear is characterized by appraisal patterns of low certainty and situational control, and thus accompanies action tendencies to reduce uncertainties [6,13]. Results from previous studies indicate that fear is positively related to the public’s risk perception during a disaster, and triggers physical action-taking to reduce risk (e.g., sheltering in place or collecting supplies) [27,29,30,36].

Anxiety is associated with a propensity for evaluation in the face of an uncertain existential threat, accompanied by a propensity to act in order to reduce this uncertainty [14,15]. Similar to fear, anxiety has a positive effect on an individual’s perception of risk and preventive behaviors [27,29]. Moreover, So et al. [37] point out that anxiety affects protection intention more than fear.

However, scholars are divided on the role of anger. Some studies suggest that anger (defined by certainty and individual control) predicts optimistic assessments, and is negatively associated with risk perception [6,7,29]. Other studies demonstrate that anger is positively associated with personal-level risk perception [15,27]. The divergence should be related to the degree of certainty and controllability associated with disaster events. Lerner and Keltner [6] pointed out that appraisals of certainty and control moderate the influence of anger on judgment and choice. When events are clearly certain and controllable (or clearly uncertain and uncontrollable), appraisal tendencies should not shape judgments; instead, valence is the most likely determinant of the emotion–judgment relationship [6]. Researchers need to specifically analyze the degree of certainty and controllability of disaster events in order to accurately judge the impact of anger on risk perception and preventive behavior.

Sadness is characterized by appraisals of loss, and thus is expected to evoke an implicit action tendency to change one’s circumstances [13]. Disgust revolves around the appraisal theme of being too close to an indigestible object or idea, and thus results in the rejection of current objects and new concepts [13,38]. Yang and Chu [27] indicate that disgust and sadness are positively related to the US public’s risk perception of the Ebola outbreak. The relationships between discrete emotions and risk perception are shown in Table 1.

## 3. Materials and Methods

### 3.1. Data Sources

This paper identifies the public’s emotions from social media users’ posts related to COVID-19 and uses the Google Shopping index of “hand sanitizer” to measure the preventive measures taken by the public.

#### 3.1.1. Social Media Data

Social media provides a convenient and inexpensive platform for the public to voice their needs. The information posted by the public reflects their positions, emotions, and opinions. This paper takes the United States as an example for quantitative analysis. Twitter was selected as the data source for the study. Twitter data from the US were derived from a dataset of COVID-19-related tweets collected by Banda et al. [39]. This dataset collected tweets containing the following keywords: “COVD19”, “CoronavirusPandemic”, “COVID-19”, “2019nCoV”, “CoronaOut break”, “coronavirus”, and “WuhanVirus”. As expected from the psychophysiological numbing phenomenon [40,41], the public’s emotional response waned as the pandemic intensified [1]. Therefore, this paper selected the data of the first year of the pandemic as the analysis object; thus, the time range of the data chosen for this paper was chosen from 21 January 2020, to 3 January 2021. All tweets are original, i.e., all retweets are removed.

This dataset provides the “country_code” field to represent the country where the tweet was written. This paper filtered tweets in the US based on the “country_code” field. However, many tweets do not provide location information, and thus the data in this paper are incomplete. The text in the tweets is multilingual, but the main language is English. The tweets selected for analysis in this paper are English tweets. The author believes this will not affect the results of the analysis. After data preprocessing, there were 1,397,032 tweets remaining with the tagged country being the United States and the language being English.

The aforementioned database only contains tweet ID, not tweet text. Hydrator [42] is the electron-based desktop application used in this paper to hydrate the Twitter ID dataset. The emotion contained in each tweet text can be obtained from the hydrated tweet text.

#### 3.1.2. Google Shopping Trends Data

The COVID-19 preventive measures recommended by public health organizations include frequent hand washing, wearing masks, etc. The public’s purchase of related commodities, such as masks and hand sanitizer, can be used as an index to measure the public’s interest in protective behaviors [43]. While the role of masks was initially controversial in the United States, experts were sure of the preventive effects of hand sanitizer from the start. Therefore, from the perspective of consistency, the purchase volume of hand sanitizer is more suitable as a measure of public preventive behavior.

**Figure 1:**
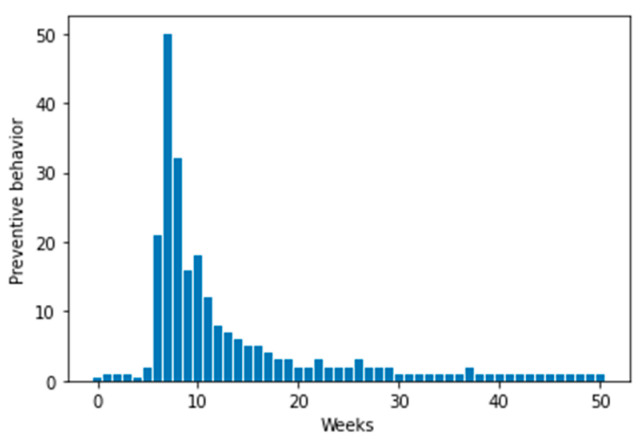
Time series of public protection behavior.

Google Trends is an analysis tool launched by Google based on search data. Google Trends can be divided into Web Search Trends, Google Shopping Trends, News Search Trends, etc. Google Shopping Trends explores the latest shopping-related search trends, and presents relative search volumes ranging from 0 to 100 (the maximum daily search volume on specific terms is standardized as 100%) [43,44]. Google Shopping indexes on hand sanitizer were used to assess the public protection behavior. Figure 1 shows the time series of hand sanitizer Google Shopping Trends data.

### 3.2. Methods

#### 3.2.1. LIWC

Texts posted by the public on social media found to be significantly associated with psychological traits can reflect individuals’ internal states, thoughts, and attitudes [45]. Anger, anxiety, and sadness were three of the most common negative emotions generated by the public during the COVID-19 pandemic [17,18,19]. Anger is an irritation-based emotion, caused by the persistence of COVID-19. Anxiety relates to fear for one’s health, safety, life, etc., caused by COVID-19. Sadness is a disappointed, sad attitude to COVID-19 and the casualties caused by it. Thus, this paper extracts three negative discrete emotions—anger, anxiety and sadness—from the text of tweets.

This paper uses Linguistic Inquiry and Word Count (LIWC for short) to analyze texts posted by the public on Twitter during the COVID-19 pandemic to identify discrete emotions. LIWC is an analysis tool that displays psychological characteristics through automated language analysis that can quantitatively analyze the word categories (especially psychological words) of text content [46,47]. LIWC operates as a text analysis program that reports the number of words belonging to a set of predefined linguistically and psychologically meaningful categories across multiple text files [1,47]. Due to the good reliability and validity of LIWC, the tool has been widely used by scholars in various fields. Moreover, it has been used to successfully identify the public’s psychological states during the COVID-19 pandemic [48].

Dictionaries, which define collections of words in particular categories, are at the heart of the LIWC program [47]. Thus, LIWC can calculate the percentage of discrete emotion words within a text, based on the collection of discrete emotion words. For example, words such as abhor*, acrimon*, affront*, aggravat*, anger*, angerer, argue, argued, arguing, argument, bad temper, bent out of shape, etc., and emoticons such as >:( are used to define anger; alarmed, alarming*, angst *, antsy, anxiet*, anxious, anxious*, apprehens*, creep*, daunt*, disquiet*, distraught*, distress*, dread, eeri*, nervous, and other words are used to define anxiety; words such as bawl*, bereav*, bittersweet*, broken heart*, broken-heart*, bummed out, cheerless*, clinical depression, crestfallen*, cried, cry, crying, deject, depress*, depressant*, clinical depression, crestfallen*, etc., and emoticons such as ): are used to define sadness. Table 2 shows some examples of discrete emotions identified from tweets using LIWC.

A set of words associated with emotion m is represented by $e_{m}$. A tweet posted by user i at time t is denoted by $t_{t}^{i}$. For a given tweet $t_{t}^{i}$, the linguistic score of emotion $p^{m}$ is the percentage of words in $t_{t}^{i}$ that belong to $e_{m}$. Table 3 shows the descriptive statistical results of emotions.

(1) $$p^{m}\left(t_{t}^{i}\right)=\frac{\left|t_{t}^{i}\cap e_{m}\right|}{\left|t_{t}^{i}\right|}\times 100$$

LIWC gives the base rate $p_{B}^{m}$ for emotion m on Twitter. To better reflect changes in public emotions caused by COVID-19, this paper uses deviations from base rates to represent anger, anxiety, and sadness. Since tweets are short documents, individual tweets will be noisy. This paper is interested in the average public emotions. Therefore, this paper groups tweets by date d and denotes these sets of tweets posted in date d as $T_{d}=\{t_{t}^{i}|t\in d\}$. Then, the linguistic score for emotion m of date d is computed as the mean of the linguistic scores of tweets in $T_{d}$, relative to the empirically observed Twitter base rate $p_{B}^{m}$:

(2) $$p^{m}\left(d\right)=\frac{100}{\left|T_{d}\right|}\sum \frac{p^{m}\left(t_{t}^{i}\right)-p_{B}^{m}}{p_{B}^{m}}$$

This paper further calculates weekly emotional linguistic scores according to the average value of the daily emotional linguistic scores and forms a time series of the weekly emotional linguistic scores, which is then used as the data source for the subsequent Granger causality test.

#### 3.2.2. Granger Causality Analysis and IRFs

The author used Granger causality analysis to answer Q1 proposed in this paper. Granger causality analysis is a statistical method of hypothesis testing that tests whether one time series is the cause of another. Granger causality analysis was conducted to determine whether emotions in a previous period (i.e., with a time lag of one, two, or three weeks) were correlated with levels of preventive behaviors in the current week, or vice versa.

This paper tested for the absence of Granger causality between variables with a vector autoregression (VAR) model, which can capture the dynamic relationships between variables. The VAR specification with the variables of this paper is shown in Model (3) below.

(3) $$\left[\begin{array}{c} \begin{array}{c} Behavior_{w}\\ Anger_{w} \end{array}\\ Anxiety_{w}\\ Sadness_{w} \end{array}\right]=\left[\begin{array}{c} \begin{array}{c} c_{1}\\ c_{2} \end{array}\\ c_{3}\\ c_{4} \end{array}\right]+\sum_{j=1}^{J}\left[\begin{array}{c} \begin{array}{c} a_{1,1}^{j}\ldots a_{1,4}^{j}\\ a_{2,1}^{j}\ldots a_{2,4}^{j} \end{array}\\ a_{3,1}^{j}\ldots a_{3,4}^{j}\\ a_{4,1}^{j}\ldots a_{4,4}^{j} \end{array}\right]\left[\begin{array}{c} \begin{array}{c} Behavior_{w-j}\\ Anger_{w-j} \end{array}\\ Anxiety_{w-j}\\ Sadness_{w-j} \end{array}\right]+\left[\begin{array}{c} \begin{array}{c} \varepsilon_{1}\\ \varepsilon_{2} \end{array}\\ \varepsilon_{3}\\ \varepsilon_{4} \end{array}\right]$$

Model (3) represents each variable as a function of its own past value, the past value of other variables, and an error term. The meanings of $Behavior_{w}$, $Anger_{w}$, $Anxiety_{w}$, and $Sadness_{w}$ are shown in Table 4. J is the maximum number of lags. $a_{i,1}^{j}\ldots a_{i,4}^{j}$ denote the coefficient matrices. $\varepsilon_{i}$ is a vector of white-noise disturbances with a normal distribution, and w is the index of a week.

The selection of the optimal lag length is based on two commonly used metrics: the Akaike Information Criterion (AIC) and the Bayesian Information Criterion (BIC). For the time series in this paper, both indexes indicated that the optimal lag length was 3.

IRFs were applied to find the impulse response between the variables and answer Q2. IRFs are able to stimulate the impact of a one-unit shock of one endogenous variable on subsequent changes in other endogenous variables, and can evaluate the importance of these changes [49]. This paper uses IRFs to assess the impact of a one-unit shock of public emotions on future changes in public protection behavior.

## 4. Results

### 4.1. Descriptive Statistics of the Data

This paper analyzes 50 weeks of US twitter texts from January 2020 to January 2021 and uses LIWC to calculate the emotional indexes of anger, anxiety, and sadness. The shopping index of hand sanitizer was collected from Google Shopping data as an index of the public’s protective behavior.

In this paper, a correlation analysis between the emotional index and the index of preventive behavior is carried out. It can be seen from Table 5 that when the significance level is 0.05, the anxiety index has a significant positive correlation with the preventive behavior index. Additionally, there is no correlation between the anger index, the sadness index, and the preventive behavior index at the 0.05 significance level.

**Figure 2:**
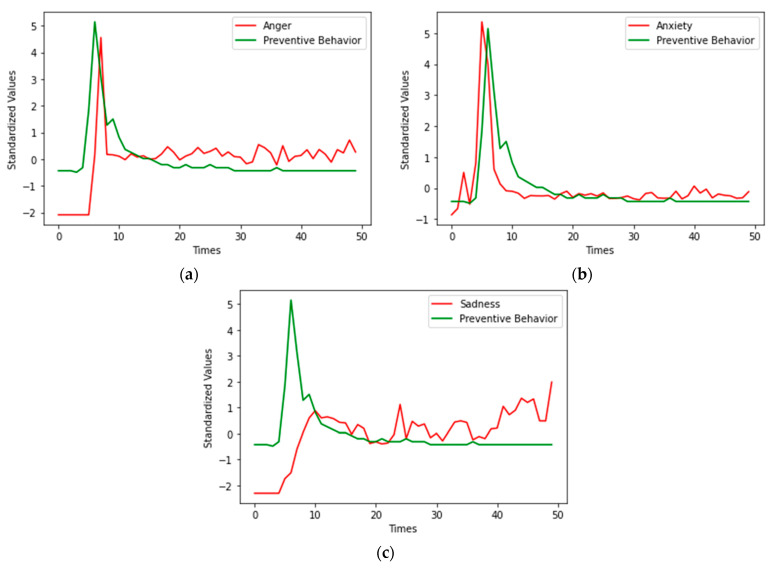
Overall emotions and preventive behaviors for 50 waves. (**a**) Anger and preventive behaviors; (**b**) Anxiety and preventive behaviors; (**c**) Sadness and preventive behaviors.

Both the emotion index and the preventive behavior index were standardized, and processed into data with a mean of 0 and a variance of 1, as shown in Figure 2. The curve trends of the anger time series and preventive behavior time series are consistent, and the curve trends of the anxiety time series and preventive behavior time series are consistent as wel; however, the curve trends of the sadness time series and preventive behavior time series are not consistent. The Granger causal relationships between public emotions and preventive behavior had to be verified with the Granger causality test.

### 4.2. Granger Causality between Discrete Emotions and Preventive Behavior

Before conducting Granger causality analysis and impulse response analysis, it is important to test the VAR model’s stationarity. After testing, the reciprocal values of all roots of the VAR model are less than 1, indicating that the structure of the VAR model is stable. As a result, the prerequisites of both Granger causality analysis and impulse response analysis are met.

Which emotions influence the public’s preventive behavior? This paper adopts the Granger causality test to determine the Granger causality between discrete emotions and preventive behaviors based on emotional time series extracted from social media posts, as well as Google Trends data reflecting public preventive behaviors.

**Figure 3:**
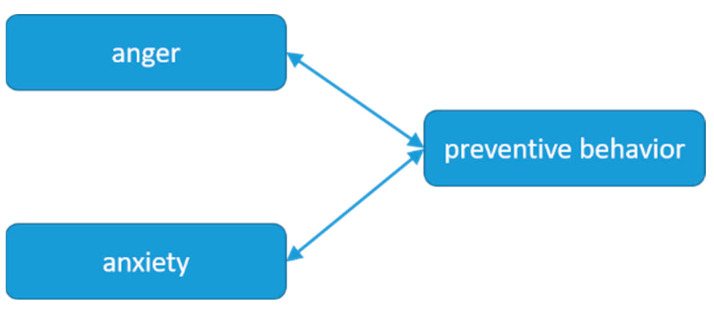
Causal channels of emotions and preventive behavior.

There is a bidirectional Granger causality between anger and preventive behavior. Anger is the Granger cause of preventive behavior, and vice versa. There is also a bidirectional Granger causality between anxiety and preventive behavior, with a 0.05 significance level and a lag of three orders. Anxiety is the Granger cause of preventive behavior, which means that the public’s anxiety affects their preventive behavior and vice versa. There is no Granger causality between sadness and preventive behavior. The results of the Granger causality tests between emotions and risk perception are shown in Figure 3 and Table 6.

### 4.3. The IRFs Results

**Figure 4:**
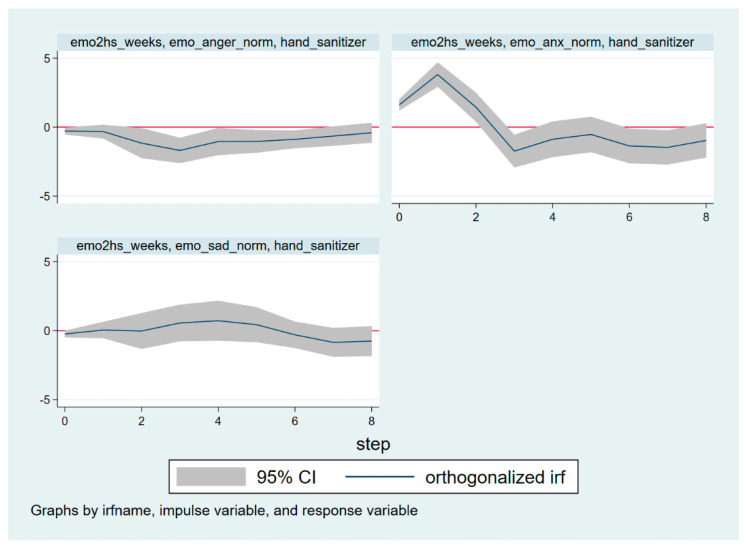
The impact of one standard deviation shock on preventive behavior.

**Figure 5:**
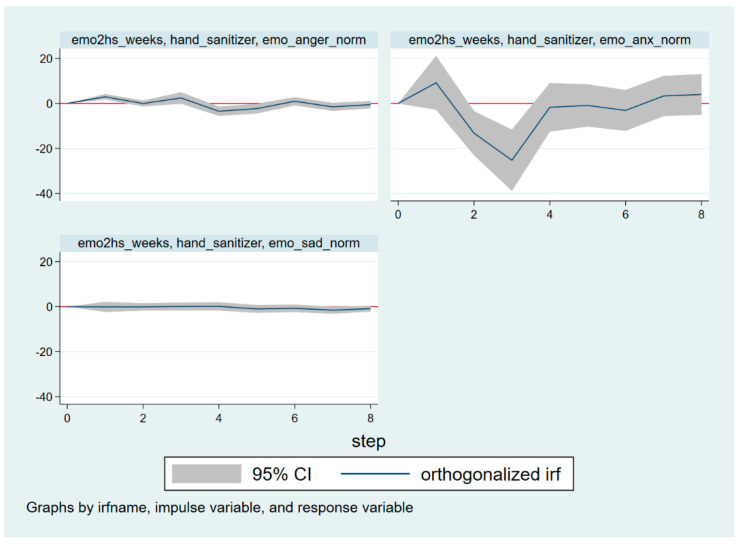
The responses of discrete emotions to one shock of preventive behavior.

This paper finds that anger Granger causes preventive behavior (*p* < 0.05), and anxiety Granger causes preventive behavior (*p* < 0.05). These results illustrate that both anger and anxiety have significant impacts on preventive behavior. Therefore, IRFs can be used to analyze the response of the preventive behavior variable in the VAR Model (3) to the disturbance of the emotional variable, and vice versa, so as to understand the dynamic characteristics of the VAR Model (3). The IRFs results are illustrated in Figure 4 and Figure 5. The *x*-axis is the timeline, which is scaled in units of time estimated by the VAR model (3). Additionally, the *y*-axis represents the response of a dependent variable to a unit of shock in the impulse variable [49]. The gray area is the 95% confidence interval.

#### 4.3.1. The Impact of Discrete Emotion Shocks on Preventive Behavior

How does a shock to discrete emotions impact preventive behavior? Figure 4 illustrates the reactions of preventive behavior to one standard deviation shock for anger, anxiety, and sadness, respectively. The horizontal axis represents the lag period (unit: weeks), and the vertical axis represents the degree of change in preventive behavior. The solid line represents the impulse response function, which represents the response of preventive behavior to the impact of each corresponding variable.

Anger had a slightly negative effect on preventive behavior. In the upper left of Figure 4, except for Time Window 3, the 95% confidence intervals basically cover the *y*-axis. This suggests that anger has a negative effect on preventive behavior at Time Window 3, while the effects at the other time windows are basically insignificant.

By observing the upper right of Figure 4, it can be seen that anxiety has a significant positive impact on preventive behavior; that is, anxiety can prompt the public to take preventive behavior. The effect of one standard deviation shock, resulting in the growth of anxiety, on preventive behavior growth is instantaneously positive from the beginning. The results also show that the maximum positive impact occurs at Time Window 1, and then the effect diminishes sharply. At Time Window 3, the effect becomes slightly negative. From Time Window 4 onwards, the 95% confidence intervals basically cover the *y*-axis, indicating that the effect of anxiety on preventive behaviors is basically insignificant. In conclusion, the effect of anxiety on prevention behavior is generally positive. Moreover, anxiety has a higher magnitude of impact on preventive behavior than anger at Time Windows 0–2.

The 95% confidence intervals cover the *y*-axis in in the bottom left of Figure 4, which suggests that the effect of one standard deviation shock to sadness on preventive behavior is not significant. This means that sadness has no significant effect on preventive behavior, which is consistent with the result of the Granger causality test.

#### 4.3.2. The Impact of Preventive Behavior Shocks on Discrete Emotions

How does a shock to preventive behavior affect different discrete emotions? Figure 5 illustrates the reactions of discrete emotions (anger, anxiety, and sadness) to one standard deviation shock of preventive behavior, respectively.

The upper left of Figure 5 shows that preventive behavior has a slightly positive effect on anger at Time Window 1, and a slightly negative effect on anger at Time Window 4. However, the magnitude of the impact was so small that it was almost negligible. In the upper right of Figure 5, except for Time Window 2–3, the 95% confidence intervals basically cover the *y*-axis. This suggests that preventive behavior has a negative effect on anxiety at Time Windows 2–3, while the effects in the other Time Windows are basically insignificant. The effect of one standard deviation shock of preventive behavior on sadness growth is not significant (in the bottom left of Figure 5).

## 5. Discussion

Previous studies have mostly used cross-sectional survey data to analyze the relationship between emotion and preventive behavior. The cross-sectional nature of the data limits the ability to make strong inferences about causal directions. That is, only the correlation between emotion and preventive behavior can be analyzed, but the causal relationship between them cannot be analyzed. This paper analyzes big data from text posted by American users on Twitter to obtain sentiment time series data. Granger causal analysis and impulse response analysis were performed on sentiment time series data and Google Trends data to identify Granger causal relationships between discrete emotions and preventive behavior, in an attempt to gain a better understanding of how public emotions influence the public to take preventive actions, and vice versa.

### 5.1. The Effects of Discrete Emotions on Preventive Behavior

Discrete emotions generate different types of propensities for action [14]. This paper analyzes the effects of three discrete emotions—anger, anxiety and sadness—on epidemic prevention behaviors.

Anger is the Granger cause of preventive behavior. The IRFs results show that the effect of anger on preventive behavior is negative, but the effect is slight. That is, anger will slightly inhibit the public from taking epidemic prevention measures. The ATF indicates that anger should generate optimistic assessments and disincentivize the public to take preventive measures against the outbreak. However, scholars are divided on the impact of anger on risk perception and consequent disaster prevention and mitigation behaviors. Some studies suggest that anger is negatively associated with risk perception [6,7,29], which is consistent with the conclusion of the ATF theory. However, other studies demonstrate that anger is positively associated with personal-level risk perception [15,27], and then triggers the public’s disaster prevention and mitigation behaviors. The results of this paper are consistent with the former. However, the results of this paper also suggest that the effect of anger is slight during COVID-19. COVID-19, as a disaster, is uncertain and uncontrollable. Lerner and Keltner [6] pointed out that appraisals of certainty and control moderate the influence of anger on judgment and choice; when events are clearly uncertain and uncontrollable, appraisal tendencies of anger should not shape judgments. Moreover, COVID-19 is a natural disaster, not a man-made one. Therefore, anger over the consequences of disasters can hardly be blamed on government departments or other humanitarian organizations. This is the reason why the conclusion of this paper is inconsistent with some studies [15,27].

The analysis in this paper shows that anxiety is also the Granger cause of preventive behavior. The IRFs results show that anxiety has a positive effect on preventive behavior. That is, anxiety will prompt the public to take epidemic-prevention measures. Anxiety, similar to fear, which is defined by great uncertainty and situational control, increases the public’s perception of risk, which in turn increases the public’s preventive behavior. According to the ATF [7], anxiety—similar to fear—should generate pessimistic assessments, prompting the public to take precautionary measures against the outbreak. The experimental results of Raghunathan and Pham (1999) [14] show that anxious individuals are biased in favor of low-risk/low-reward options. Anxiety is one of the central motivating forces fueling the intention to take protective actions [27,29,37]. The conclusion of this paper is consistent with these studies. The IRFs results also show that anxiety is more important than anger in shaping preventive behavior. So, using survey data, Kuang and Cho [37] point out that anxiety affects protection intention more than fear. These conclusions suggest that anxiety is the most important emotion affecting public risk perception and protective behavior during crises. Crisis communicators should pay special attention to public anxiety when communicating with the public.

Sadness is not the Granger cause of preventive behavior. The IRFs results are also consistent with this. Studies have shown that sad individuals are biased in favor of high-risk/high-reward options [14]. However, Yang and Chu [27] indicate that sadness is positively related to the US public’s risk perception of the Ebola outbreak, while the results of this paper show that sadness has no effect on public protective behavior in health crises.

The results of Granger causality analysis and IRFs confirm that affective states of the same valence may have distinct influences on protective behavior during emerging health threats. In practical terms, our findings suggest that public health communicators and policymakers should pay more attention to the different roles of discrete emotions in preventive behavior during emerging health threats. Understanding the impact of discrete emotions on prevention behaviors can help researchers understand the extent of the cooperation of the public in mitigating public health risks.

### 5.2. The Effects of Preventive Behavior on Discrete Emotions

Preventive behavior is the Granger cause of anger. However, the IRFs results suggest that the effect of preventive behavior on anger is very small and negligible. Preventive behavior is the Granger cause of anxiety. The IRFs results show that the effect of preventive behavior on anxiety is negative, and the influence has a certain lag. This indicates that the adoption of preventive behaviors can bring certain comfort to the public and alleviate the public’s anxiety, but the effect is not immediate and there is a lag period. Preventive behavior is not the Granger cause of sadness. The IRFs results are also consistent with the results of the Granger causality analysis. These results suggest that preventive behavior has little effect on public anger and sadness.

Compared with the literature on the influence of emotion on protective behavior, there are relatively few studies on the influence of protective behavior on emotion. Peters et al. [50] point out that risk perception is a function of negative emotion rather than vice versa. However, through the Granger causality test and IRFs, this paper finds that preventive behaviors can alleviate public anxiety with a certain lag. This can help public health managers and other stakeholders communicate more effectively with the public. Tracking public emotions through social media monitoring can be useful when communicating with ordinary citizens during public health crises [15].

### 5.3. Limitations and Future Work

The method proposed in this paper can be easily applied to other disaster events; however, the results may not be universal. In fact, the behavior of the public is influenced by the nature of disaster, which may cause the relationship between discrete emotions and disaster response behavior in other disaster events to be inconsistent with the conclusions of this paper. When the controllability and certainty of disaster events are ambiguous, the differential evaluation tendency of discrete emotions is most significant [6,27]. Exploring a wider range of disaster events that vary in certainty and controllability can improve our understanding of the interplay between different discrete emotions and public preventive behaviors.

One limitation of this study is that we only used Twitter data to measure the public’s emotions. Due to the existence of digital inequality, some vulnerable groups may lack representation or fail to speak online [51,52,53]. This research is based on social media data, which may not adequately reflect the emotions of vulnerable groups, such as the elderly, who were in fact more affected by the pandemic. How to combine online and offline data to fully reflect the emotions of the public, especially vulnerable groups, is a potential direction of further research.

Another limitation is the representativeness of the Twitter data used in this paper. First of all, Twitter is not a mainstream social media in some countries or regions, such as China. Secondly, less than 1% of all tweets are actually geo-tagged, which will lead to issues of over- or underrepresented populations [54]. Thirdly, people may use a VPN to change their location, so some tweets may not actually be from the US. These factors could result in imprecision in the results of this paper.

## 6. Conclusions

Based on the textual analysis of tweets from the United States, this paper assesses the Granger causal relationships between three discrete negative emotions (anger, anxiety, and sadness) and pandemic-preventive behavior. Anger is the Granger cause of preventive behavior and has a slightly negative effect on the public’s preventive behavior. Anxiety is the Granger cause of preventive behavior and has a positive effect on preventive behavior. We can identify how different emotions play qualitatively distinct roles in promoting preventative behaviors by examining how discrete emotions such as anger and anxiety affect these actions. Moreover, preventive behavior is the Granger cause of anxiety and has a negative and lagging effect on anxiety.

Public health communicators and policymakers should pay more attention to the role of emotions during public health crises. Considering the role of emotion is one of the components of a coordinated communication endeavor. By focusing on the interplay between protective behaviors and emotional responses, we hope to gain a deeper understanding of the impact of discrete emotions on people’s risk-related choices, and vice versa. Understanding the impact of discrete emotions on preventive behaviors can help the extent of cooperation on the public’s part to mitigate public health risks. On the other hand, understanding the impact of prevention behaviors on discrete emotions is useful for better risk communication.

## Figures and Tables

**Table 1:** The relationships between discrete emotions and risk perception.

Discrete Emotion	Positive Effect	Negative Effect	Reference
Fear	True		[27,29,30,36]
Anxiety	True		[27,29,37]
Anger		True	[6,7,29]
Anger	True		[15,27]
Disgust	True		[27]
Sadness	True		[27]

**Table 2:** Examples of discrete emotions identified from tweets using LIWC.

No.	Tweet Text	Discrete Emotion
1	COVID-19 is starting to piss me off	Anger
2	Hate, hate, hate, double hate, LOATHE ENTIRELY	Anger
3	Fear. Fear. Fear. #COVID19	Anxiety
4	I’m stressed and overwhelmed #COVID19	Anxiety
5	Cry cry cry	Sadness
6	uhhhhhhhh ohhhhh :( :( :( :( #COVID19	Sadness

**Table 3:** Descriptive statistics of emotions.

Emotion	Minimum Value	Maximum Value	Mean	Standard Deviation
Anger	0.00	42.86	0.08	0.76
Anxiety	0.00	75.00	0.12	0.86
Sadness	0.00	50.00	0.11	0.93

**Table 4:** Definition of variables.

Variable	Definition
$Behavior_{w}$	Preventive behavior, expressed by Google Shopping Index within time window w
$Anger_{w}$	The index of anger within time window w
$Anxiety_{w}$	The index of anxiety within time window w
$Sadness_{w}$	The index of sadness within time window w

**Table 5:** The correlation coefficient between the emotion index and preventive behavior index.

Emotion	Correlation Coefficient	*p*-Value
Anger	0.2716	0.0564
Anxiety	0.7082	0.0000
Sadness	−0.2132	0.1371

**Table 6:** Results of Granger causality tests between emotions and preventive behavior in the US.

Null Hypothesis	Lag	F-Value, *p*-Value	Results
Anger is not the Granger reason for preventive behavior.	3	10.133, 0.017	Reject
Anxiety is not the Granger reason for preventive behavior.	3	194.92, 0.000	Reject
Sadness is not the Granger reason for preventive behavior.	3	5.0919, 0.165	Accept
Preventive behavior is not the Granger reason for anger.	3	32.277, 0.000	Reject
Preventive behavior is not the Granger reason for anxiety.	3	32.277, 0.003	Reject
Preventive behavior is not the Granger reason for sadness.	3	3.1944, 0.363	Accept

## Data Availability

The data presented in this study can be downloaded at: https://zenodo.org/record/4414856#.Y0ktlUfP1BA (accessed on 6 November 2022).
